# Professor Bartlett

**Published:** 1846-08

**Authors:** 


PROFESSOR BARTLETT.

The Western Lancet, referring to the appointment of Dr. Bartlett as Professor of the Theory and Practice of Medicine in Transylvania University, says, "his last letter contains a formal acceptance of the Chair." He is expected in Lexington before the opening of the lectures in November. The Lancet speaks of his health as being good.

